# Assessing Cognitive Function in Neuromuscular Diseases: A Pilot Study in a Sample of Children and Adolescents

**DOI:** 10.3390/jcm10204777

**Published:** 2021-10-18

**Authors:** Rossella D’Alessandro, Neftj Ragusa, Martina Vacchetti, Enrica Rolle, Francesca Rossi, Chiara Brusa, Chiara Davico, Benedetto Vitiello, Tiziana Mongini, Federica S. Ricci

**Affiliations:** 1 Section of Child and Adolescent Neuropsychiatry, Department of Public Health and Pediatric Sciences, University of Turin, 10126 Turin, Italy; rossella.dalessandro@unito.it (R.D.); martina.vacchetti@unito.it (M.V.); enrica.rolle@gmail.com (E.R.); francesca.rossi@unito.it (F.R.); chiara.brusa@unito.it (C.B.); chiara.davico@unito.it (C.D.); benedetto.vitiello@unito.it (B.V.); 2 Section of Pediatrics, Department of Public Health and Pediatric Sciences, University of Turin, 10126 Turin, Italy; neftj.ragusa@unito.it; 3 Neuromuscular Unit, Department of Neurosciences, University of Turin, 10126 Turin, Italy; tizianaenrica.mongini@unito.it

**Keywords:** neuromuscular disorders, cognitive functioning, motor functioning

## Abstract

Central nervous system (CNS) involvement has been variously studied in pediatric neuromuscular disorders (NMDs). The primary goal of this study was to assess cognitive functioning in NMDs, and secondary aims were to investigate possible associations of cognitive impairment with motor impairment, neurodevelopmental delay, and genotype. This was a cross-sectional study of 43 pediatric patients, affected by six NMDs. Myotonic dystrophy type 1 (DM1) and glycogen storage disease type 2 (GSD2) patients had a delay on the Bayley-III scales. On Wechsler scales, DMD and DM1 patients showed lower FSIQ scores, with an intellectual disability (ID) in 27% and 50%, respectively. FSIQ was normal in Becker muscular dystrophy (BMD), GSD2, and hereditary motor sensory neuropathy (HMSN) patients, while higher individual scores were found in the spinal muscular atrophy (SMA) group. In the DM1 cohort, lower FSIQ correlated with worse motor performance (ρ = 0.84, *p* < 0.05), and delayed speech acquisition was associated with ID (*p* = 0.048), with worse cognitive impairment in the congenital than in the infantile form (*p* = 0.04). This study provides further evidence of CNS in some NMDs and reinforces the need to include cognitive assessment in protocols of care of selected pediatric NMDs.

## 1. Introduction

The last 20 years have witnessed a major expansion in the research and clinical management of neuromuscular disorders (NMDs), with an exponential increase in the knowledge of the basic biological-molecular mechanisms of illness and the introduction of innovative therapeutic applications. Considerable progress has been made in the development of causal, mechanism-based, and disease-modifying therapies, especially with the innovative application of genetic therapy technologies. For Duchenne muscular dystrophy (DMD) patients, steroids are now part of the recommended standard of care [1], and genetic therapies are available for some patients (e.g., ataluren, an oral drug that allows stop codons readthrough to take place for patients with premature stop codon point mutations [2], and eteplirsen, an antisense oligonucleotide for patients amenable to exon 51 skipping [3]). Since 2006, the standard care of glycogen storage disease type 2 (GSD2, or Pompe disease) has been enzyme replacement therapy (ERT) [4,5]. For spinal muscular atrophy (SMA) patients, three breakthrough disease-modifying therapies have been approved: nusinersen and risdiplam, which are based on antisense oligonucleotides (ASO) technologies, and an adeno-associated virus (AAV9)-mediated gene therapy (onasemnogene abeparvovec). To date, no causal drug is available for other NMDs such as myotonic muscular dystrophy type 1 (DM1), hereditary motor sensory neuropathy (HMSN, also known as Charcot–Marie–Tooth disease, CMT) and Becker muscular dystrophy (BMD); however, also in these NMDs the ameliorated care protocols have significantly prolonged the survival.

The new disease-modifying therapies, associated to appropriate standard of care, are improving the prognosis of patients and leading to the emergence of new clinical phenotypes [6], with most NMDs showing features of multisystemic disorders [1]. Data are becoming available about the involvement of the central nervous system (CNS), especially during the developmental age, not only concerning the aspects directly related to the underlying neuromuscular dysfunction, but also regarding the development of the cognitive and neuropsychological profile [7,8,9].

Cognitive impairment, apparently independent from motor impairment [10], is recognized as a feature in about one third of DMD patients [7], and delay in global or language development can constitute an early sign of this disease [10]. In a meta-analysis of more than 1000 patients with DMD or BMD, the mean full-scale IQ (FSIQ) was one standard deviation below the population mean, but with scores ranging from severe impairment to above average [11]. A disproportionate effect on verbal working memory skills has also been found [11,12], with a dissociation between verbal and performance intelligence quotients (VIQ and PIQ) [13]. Moreover, it has been increasingly reported that in DMD patients CNS involvement could be associated with symptoms of neurodevelopmental disorders, with higher prevalence of autistic spectrum disorder (ASD), attention-deficit–hyperactivity disorder (ADHD), and obsessive–compulsive disorder [7]. Even if great variability in intellectual functioning was reported by several early studies, the reason was not clearly understood until very recently. The dystrophin (Dys) gene contains 79 exons plus seven internal promoters that generate a range of different protein isoforms, with diverse expression in tissues. The full-length Dys isoforms (Dp427M, Dp427C, Dp427P) are largely neuronally expressed, and play an important role in the architectural organization of the CNS, being involved in the organization of GABAergic synapses in the cortex, hippocampus, and cerebellum [12]. Even if the role of Dys isoforms in the brain is still largely unknown, it is recognized that distal mutations disrupting the brain isoforms Dp140 and Dp71 are more frequently associated with lower FSIQ scores [7,14,15,16], and with reduced grey matter volume [17]. In contrast, in individuals with mutations affecting only the full-length isoforms (upstream of exon 30), only a minimal frequency of intellectual impairment is observed [12].

Little is known about the CNS involvement in BMD, and the type and prevalence of cognitive impairment has not been determined, even though these patients appear to have average cognitive functioning, with few reports of a slight reduction in verbal skills [8], learning difficulties, and behavioral problems [18,19].

CNS involvement is a core manifestation of DM1 [8,20], but there is high between-study heterogeneity [21]. In congenital DM1, moderate to severe intellectual disability (ID) has been described with involvement across different domains; nevertheless some congenital patients show normal intellectual functioning and brain structure [21]. In childhood DM1, mild to moderate ID with impairment of visuospatial skills have been reported [20,22,23]. A negative correlation between cytosine-thymine-guanine (CTG) expansion size and cognitive function has been reported [24,25,26], with more marked intellectual deficits in patients with larger CTG repeats compared to those with shorter expansion [20,27,28]. A cognitive decline over time, or a slower rate of development with a tendency to cognitive decline, has been reported, but, until now, the pathophysiology and temporal development of brain involvement still remain unclear [21].

Patients with infantile-onset Pompe disease (IOPD) are now living longer since the advent of intravenous (ERT) [6]. Brain autopsies of untreated infants showed widespread glycogen storage in the CNS [29], but this therapy is unlikely to cross the blood–brain barrier, thus increasing concerns about long term effects on CNS [29,30]. Ebbink and Spiridigliozzi [29] suggested that, in children with GSD2 treated with ERT, despite the strong evidence of glycogen storages in the brain, the impact on CNS seems to be limited. On the other hand, they found that patients who started ERT early had the best motor outcome and the highest scores on early cognitive development. A great cognitive variability emerged in one long term study of children with IOPD treated with ERT [31].

Concerning patients affected by SMA, with the increasing number of long-term survivors worldwide, new phenotypes are arising, suggesting that some patients may show cognitive impairment, in particular those with SMA type 1. Clinical practice shows that most of these untreated children never achieve functional verbal skills [32,33]. Poor cognitive performance was more frequently reported in two recent studies of cognition that specifically targeted patients with SMA type 1 [34,35]. 

Regarding HMSN, since it is a peripheral neuropathy, there is almost no literature concerning CNS involvement. A recent study published in 2017 has investigated a possible role of PMP22 protein (lacking in CMT1A) in CNS functioning: results of this study suggest that an altered expression of PMP22 determines a mild, but significant, ID and a reduction in the volume of white matter on MRI, compared to healthy subjects, but further studies in pediatric age are needed [36].

The introduction of new treatments and the consequent improved survival and improvement in motor functions make the assessment of cognitive functioning especially timely and clinically relevant to the management of subjects with NMDs. This pilot study aimed to test the feasibility of evaluating CNS involvement through the assessment of neurocognitive functioning in children affected by genetic NMDs of different etiology and degrees of motor impairment. Possible associations between motor deficit and intellectual functioning, developmental delay and cognitive impairment, and genotype–phenotype correlations were also investigated. The study was intended as an initial step towards ultimately characterizing cognitive phenotypes and improving clinical management.

## 2. Materials and Methods

### 2.1. Study Design

This was a single-center, observational, cross-sectional study conducted at the child neuropsychiatry services of the University of Turin Pediatric Hospital “Regina Margherita”, Turin, Italy, over a 6-month period. Cognitive functioning was assessed in children and adolescents affected by 6 of the main NMDs followed at our center: DMD, BMD, DM1, GSD2, SMA type 2 and 3, and HMSN type 1 and 2. The study was approved by the institution’s research ethics committee and written informed consent to study participation was provided by parents or legal guardians.

### 2.2. Participants and Procedures

Patients aged below 17 years, with a diagnosis of NMDs made within the first 12 years of life, were enrolled. NMDs were diagnosed according to the current international standard criteria, based on clinical and laboratory characteristics with genetic-molecular confirmation.

Patients were excluded if unable to adequately perform the whole cognitive assessment because of too severe disabling motor impairment or if unable to understand instructions during tests for linguistic reasons (i.e., non-Italian speaking patients).

Cognitive and motor function assessment scales, appropriate for age and pathology, were administered. Patients who had not undergone a cognitive assessment during their clinical history were recalled and assessed during the study period, while for those previously assessed, the results were retrieved retrospectively. Patients’ motor skills data were collected at follow-up visits, and parents or caregivers were interviewed for neonatal and neurodevelopment information. Assessments were administered and scored by trained child and adolescent neuropsychiatrists and by neuropsychomotor rehabilitation therapists.

### 2.3. Assessments

#### 2.3.1. Cognitive Functioning

Cognitive assessment was conducted using standardized instruments, not yet specifically validated for NMDs, but universally recognized as reference points for developmental and cognitive assessment in developmental age. For children aged between 1 and 42 months, the Italian validated version of Bayley Scale of Child and Infant Development Third Edition was performed [37].

The Italian standardization of the Wechsler scales ([38]) was used and, depending on the age of the patient, the appropriate scale was performed to assessed global intelligence measure, in particular:

- Wechsler Preschool and Primary Scale of Intelligence, Third Edition (WPPSI-III), for children aged between 2 years and 6 months and 7 years and 3 months.
- Wechsler Intelligence Scale for Children, Fourth Edition (WISC-IV), for patients aged between 6 years and 16 years and 11 months.

The Wechsler scales are standardized tests that yield an FSIQ, representing overall intellectual ability, and index scores that measure different domains such as verbal intelligence, non-verbal intelligence, working memory and processing speed. WPSSI-III sub-indices are VIQ, PIQ and processing speed quotient (PSQ), while WISC-IV sub-indices are verbal comprehension index (VCI), perceptual reasoning index (PRI), working memory index (WMI) and processing speed index (PSI).

#### 2.3.2. Comparator Group for Cognitive Assessment

We used the Italian standardized normative data provided in the Wechsler IQ tests for comparison. Both the WISC-IV and WPPSI III have large normative data that are reported to be representative of the Italian speaking population and are stratified by age, gender, ethnicity, education level, and geographical region. 

The details of normal population are as follows:

- WISC-IV: *n* = 2200 subjects (1100 males and 1100 females), aged between 6–16 years, 11 months, and 30 days, attending primary, middle and high-school.
- WPSSI-III: *n* = 987 subjects, aged 2 years and 6 months–7 years and 3 months, attending public school and kindergarten, proportioned to the Italian general population.

The normative population mean across both measures is 100 with a standard deviation of 15 for sub-indices scores and have a mean of 10 with a standard deviation of 3 at the subtest level.

#### 2.3.3. Motor Function Assessment

Motor function assessment was performed using standardized rating scales, many of which have already been validated for NMDs, and are used in usual practice.

All the scales are described and are summarized in Table S1. 

### 2.4. Data Analysis

Descriptive analyses were performed for all the demographic and clinical variables of interest (categorical variables were expressed as frequencies and percentages, and continuous variables as median and IQR, or mean and SD when appropriate).

Concerning analyses on cognitive profile, all assessment instruments described above have been standardized for use in Italy and, therefore, the mean and SD for each subscale in a normative population is known. Consequently, for DMD, BMD, DM1 and HMSN subgroups, given that a difference with normal distribution could not be found with a Shapiro–Wilk test for FSIQ and sub-indices distribution, the one-sample Z-test was applied to compare FSIQ and sub-indices mean scores with normative data provided in the Italian validated version of Wechsler IQ scales. Descriptive results data were provided for SMA patients group.

When appropriate, Pearson’s simple linear correlation analyses were used to investigate possible associations between motor function scores and cognitive performance within NMD subgroups. 

In both DMD and DM1 cohorts, the Fisher exact test was run to investigate a possible association between ID (i.e., FSIQ < 70) and a delayed walking acquisition (i.e., age autonomous walking acquisition ≥ 18 months), and between the presence of ID and a delayed language development (i.e., first words pronounced after 12 months of age). 

In the DMD sample, *t*-test was used to evaluate the relationship between the location of the mutation in the DMD gene (proximal or distal) and the WISC-IV and WPSSI-III FSIQ scores together. Moreover, in DM1 patient cohort, the *t*-test was performed to investigate possible FSIQ score differences on WISC-IV scale between the congenital and the infantile form. 

For statistical analyses, a two-tail significance level α of 0.05 was set; regarding FSIQ and sub-indices on Wechsler scales, comparative analyses with normative population data, a Bonferroni correction for multiple comparisons was applied, giving an α of 0.01 (five tests) for WISC-IV, and a α of 0.0125 WPSSI-III (four tests). 

Statistical processing was performed using the IBM SPSS Statistic software, version 24 (IBM Corp., Armonk, NY, USA).

## 3. Results 

The sample included a total of 43 pediatric patients: 15 subjects affected by DMD, 4 by BMD, 8 by DM1, 6 by GSD2, 3 by type 2 or 3 SMA, and 7 by type 1 or 2 HMSN.

### 3.1. Sample Demographics, Clinical and Genetics Characteristics

Patients’ full demographics and clinical characteristics are summarized in Table 1.

### 3.2. Cognitive Assessment and Correlation Analyses

#### 3.2.1. Duchenne Muscular Dystrophy (DMD)

Fifteen DMD patients, all male, with a median age at assessment of 6.7 years (IQR 4.3, range 4.1–13.7 years) were recruited.

Twelve subjects (80%) had a wide out-of-frame deletion in the locus Xp21 dystrophin gene, one patient had an out-of-frame duplication of exon 44, and two patients (13%) had a point mutation within exon 43.

Cognitive function was assessed with WPPSI-III (*n* = 7), or WISC-IV (*n* = 8) based on the patient’s age. FSIQ was mean 96.9 (SD 26.1) on WPSSI-III scales, and 75.9 (SD 11.1) on WISC-IV scales, with no significant difference between the two FSIQ means (*t*-test *p* = 0.08). Eleven patients (73%) had an FSIQ score on WPPSI-III or WISC-IV scales greater than 70, in particular 4 (36%) were in the borderline range (i.e., FSIQ between 70 and 84) and 2 (18%) in the high range (i.e., FSIQ > 115, 128 and 132 respectively) compared to normative data for age. Four subjects (27%, *n* = 1 at WPPSI-III and *n* = 3 at WISC-IV) had an FSIQ lower than 70, presenting an ID of mild degree. No case of moderate or severe ID (i.e., FSIQ score lower than 50) was found in this cohort. 

Compared to the normative population FSIQ mean scores, WISC-IV FSIQ mean score was significantly lower (mean 75.9, SD 11.1, Z −4.54, *p* < 0.0001), while the WPPSI-III FSIQ mean score was not significantly different (mean 96.9, SD 26.1, Z −0.58, *p* = 0.56).

**Figure 1:**
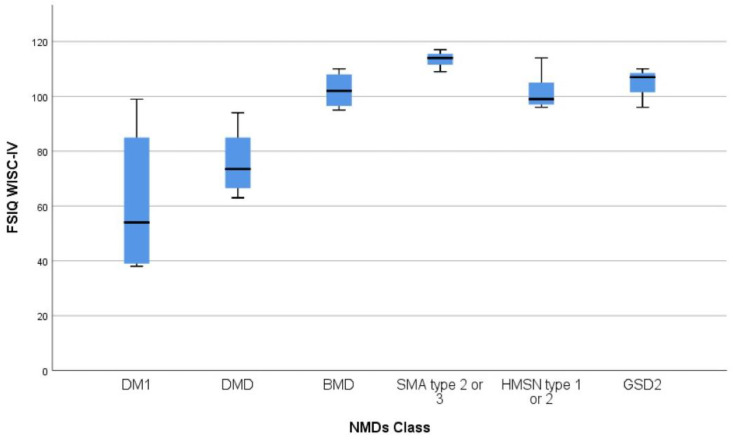
WISC-IV FSIQ boxplots across NMDs cohorts. WISC-IV: Wechsler Intelligence Scale for Children—Fourth Edition; FSIQ: Full Scale IQ; NMDs: Neuromuscular Disorders; DM1: myotonic muscular dystrophy type 1; DMD: Duchenne muscular dystrophy; BMD: Becker muscular dystrophy; SMA: spinal muscular atrophy, type 2 or 3; HMSN: hereditary motor sensory neuropathy, type 1 or 2; GSD2: glycogen storage disease type 2.

On the WISC-IV scale, all the four sub-indices mean scores resulted significantly lower compared to the normative mean scores (VCI mean 78.7, SD 7.7, Z −4.0, *p* < 0.00001, PRI mean 82.9, SD 8.4, Z −3.2, *p* < 0.0013, WMI mean 80.9, SD 9.7, Z −3.6, *p* = 0.0003 and PSI mean 82.2, SD 14.2, Z −3.3, *p* = 0.00078) (Table 2, Figure 1).

On the WPPSI-III scales, all the four sub-indices mean scores were not significantly different compared to normative mean scores (VIQ mean 91.7, SD 22.9, PIQ mean 95.7, SD 21.2, PSQ mean 85.6, SD 20.9, all *p* > 0.01).

**Figure 2:**
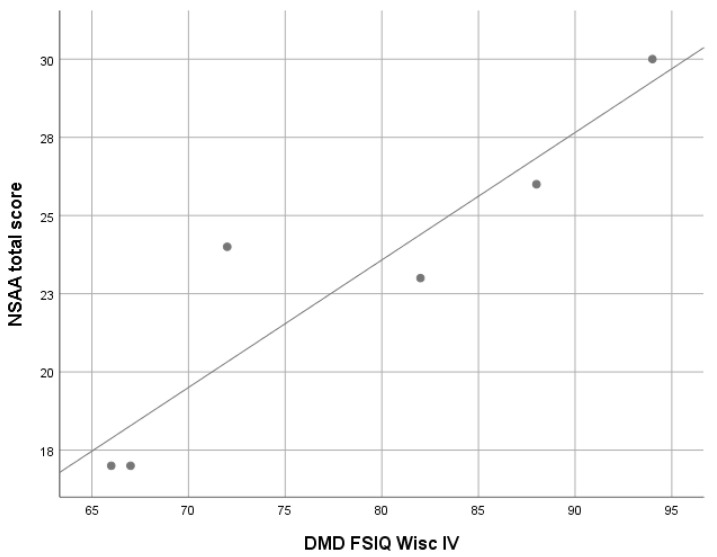
Correlation analysis between WISC-IV FSIQ and NSAA total scores in Duchenne muscular dystrophy (DMD) cohort. WISC-IV: Wechsler Intelligence Scale for Children, Fourth Edition; FSIQ: Full Scale IQ; NSAA: North Star Ambulatory Assessment Scale; Linear R^2^ = 0.854.

A significant correlation between WISC-IV FSIQ scores and North Star Ambulatory Assessment (NSAA) total scores was found (ρ 0.92; *p* = 0.008), while no significant correlations were found between the 6-minute walking test (6MWT) total scores and WPPSI-III or WISC-IV FSIQ scores (Figure 2).

Since in literature there is growing evidence that intellectual functioning in DMD patients is variable depending on whether the mutations are located proximally or distally within the Dys gene, with a greater negative impact for more distal mutations, association analyses were performed to investigate a possible difference in this cohort. The cut-off, to discriminate between proximal and distal, was established by the Authors prior to exon 30 [39] or exon 45 [40], depending on the case. By establishing exon 30 as the cut-off, only two patients have a proximal mutation and 13 a distal mutation, thus exon 45 was considered as the cut-off to run analyses. On this basis, 8 (53%) patients have a proximal mutation, and 7 (46%) have a distal one. Considering WISC-IV and WPPSI-III FSIQ together, in the group with a proximal mutation the FSIQ mean score was 87.0 (SD 20.9), while in the group with a distal mutation it was 85.1 (SD 23.8), with no significant difference between the two compared groups (*t*-test, *p* > 0.05). 

No significant associations emerged between a history of delayed language or walking acquisition and the presence of ID at assessments (Fisher exact test *p* = 0.08 in both cases).

#### 3.2.2. Becker Muscular Dystrophy (BMD)

Four patients, all male, with a median age at assessment of 12.1 years (IQR 6.1, range 8.7–15.7 years) were enrolled. 

All patients had in-frame deletions in the dystrophin gene. Two subjects had never presented neuromuscular symptoms, while in the other two the disease started with fatigue and cramps/rhabdomyolysis at 3 and 6 years of age respectively (Table 1). 

FSIQ and sub-indices scores, assessed with WISC-IV scales in all four patients, were in the normal range for age in all cases, with no significant differences compared to normative data (Z-test, *p* > 0.01 in all cases) (Table 2, Figure 1).

#### 3.2.3. Myotonic Dystrophy Type 1 (DM1) Patients

Eight patients, 4 males, with a median age at assessment of 12.9 years (IQR 6.6, range 1.5–16.4 years) were assessed.

Cognitive assessment was performed with the Bayley-III (*n* = 2) or WISC-IV scales (*n* = 6), based on the patient’s age.

On the Bayley-III, on the Cognitive Scale one patient had a delay compared to normative data, while the other scored at the lower limits of the norm. Language Scale scores were lower in both children compared to the normative population scores for age, with one also showing a delay on the Motor Scale. It is noteworthy that one child was bilingual.

Considering WISC-IV scales results, FSIQ mean score was significantly lower compared to the normative data mean (mean 61.5, SD 26.1, Z −6.36, *p* < 0.00001), with ID in four out of six patients, of mild degree in one patient and moderate in the other three. Of the two patients with a FSIQ score greater than 70, in one case the FSIQ score was at the lower limits of the norm for age. All WISC-IV sub-indices mean scores were significantly lower compared to the normative population mean score (at Z-test all *p* < 0.00001). (Table 2, Figure 1).

**Figure 3:**
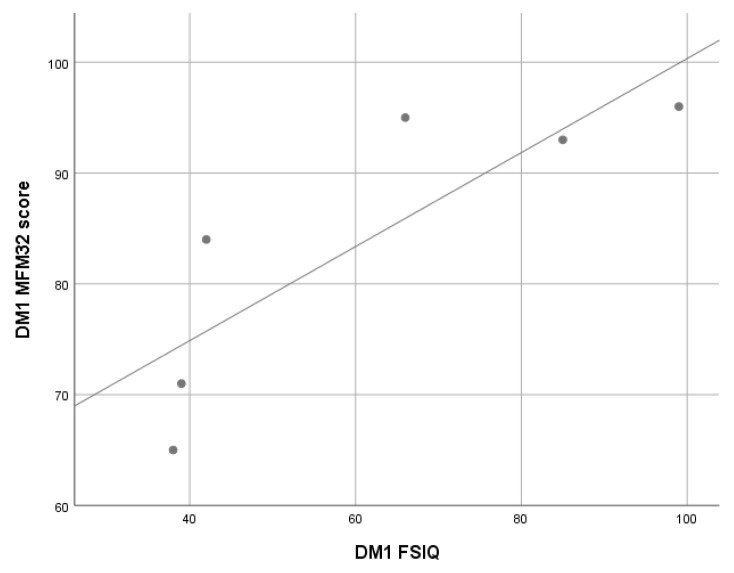
Correlation analysis between WISC-IV FSIQ and total scores on the MFM-32 in myotonic dystrophy type 1 (DM1) cohort. WISC-IV: Wechsler Intelligence Scale for Children, Fourth Edition; FSIQ: Full Scale IQ; MFM32: Motor Function Measure—32 items; Linear R^2^ = 0.072.

Correlation analyses showed that, in this cohort, WISC-IV FSIQ scores were significantly associated with the total scores on the Motor Function Measure—32 items (MFM-32) scale (ρ 0.84; *p* < 0.05), while no significant correlation was found between 6MWT scores and WISC-IV FSIQ scores (Figure 3).

Moreover, in this cohort, a significant association emerged between a history of delayed language acquisition and the presence of ID at the study assessment (Fisher exact test *p* = 0.048), while no association emerged with a history of delayed walking acquisition.

Based on previous reports [20,27], it was also investigated whether there was an association between the DM1 phenotype (i.e., congenital form, *n* = 3, or infantile form, *n* = 3) and the degree and frequency of cognitive impairment: a greater severity of cognitive impairment was found in the congenital form compared to the infantile one (*t*-test, *p* = 0.04). 

#### 3.2.4. Glycogen Storage Disease Type 2 (GSD2) Patients

Six patients, 4 males, with a median age at assessment of 8.4 years (IQR 13.0, range 1.7–16.9 years) were enrolled. 

Two patients were affected by the classical infantile form of GSD2 disease, while 4 by the later onset form. At the time of assessment four patients were being treated with ERT, while two, both affected by the later onset form and asymptomatic, were not receiving ERT.

The two patients assessed with the Bayley-III scales scored in the normal range for age on the cognitive scale, with an associated fall on the language scale (4^°^ and 6^°^ percentile rank respectively) in both, while remaining in the normality range for age. One patient had low scores for age in the motor area (2° percentile rank), with a greater involvement of gross motor skills, and in Adaptive Behavior Scale (2° percentile rank).

FSIQ and sub-indices scores assessed by WISC-IV, were in the normal range for age in all three cases assessed, with no significant differences compared to normative data (Z-test, *p* > 0.01 in all cases) (Table 2, Figure 1).

#### 3.2.5. Spinal Muscular Atrophy (SMA) Patients (Type 2 e 3)

Three SMA patients, two with type 2 and one with type 3, 2 males, with a median age at assessment of 10.7 years (IQR 1.0, range 10.2–11.2), were enrolled.

Cognitive function was assessed by WISC-IV scales in all three subjects showed. FSIQ median score was 114.0 (IQR 5.0), and, in particular, FSIQ score was in the normal range in two patients and in the higher range compared to population data for age in the remaining one (Table 2, Figure 1). 

Concerning WISC-IV sub-indices scores, for all three patients the PSI score was in the normal range, with a median of 97.0 (IQR 1.5), while the VCI score was a point of strength, with a median of 118.0 (IQR 1.0). In one patient also the PRI was a strength (PRI index score 119).

#### 3.2.6. Hereditary Motor Sensory Neuropathy (HMSN) Patients

Five patients, 2 males, with a median age at assessment of 11.5 years (IQR 5.8, range 4.2–14.3 years), were enrolled. Two patients were affected by type 1A form, two patients (brothers) by type 1B, while one patient was affected by type 2A form (Table 1). 

Cognitive function in this cohort was examined by WPPSI-III scales (*n* = 1) and WISC-IV scales (*n* = 6). The WISC-IV FSIQ mean score was 101.7 (SD 6.2), with all subjects having a FSIQ score in the normal range. FISQ and sub-indices mean scores were not significantly differentce compared to normative data (Z-test, *p* > 0.01 in all cases) (Table 2, Figure 1). 

Individual scores for each NMD are reported in Tables S2–S4.

## 4. Discussion

In this study, the global intellectual functioning of children with NMDs of different etiology was systematically assessed and compared with the Italian normative population data for age provided by Wechsler IQ scales. Furthermore, to investigate possible associations with the degree of motor impairment, in the whole sample the motor function was systematically measured through validated motor performance tests. 

Because of the low population prevalence of NMDs, the study sample size was rather small for precise statistical estimates, and there was numerical heterogeneity among NMD subgroups, patients age and, therefore, among cognitive and motor function assessment scales used. However, despite these limitations, several inferences can be drawn. 

In general, it is not easy to obtain a standardized assessment of intelligence in children with neuromuscular disabilities, especially when severe. In fact, many of the standard cognitive tests for older children are based on general knowledge and experience, which can be limited precisely because of motor disabilities. Furthermore, many tests depend on motor ability and speed of manipulation and coordination, abilities that can be reduced by muscle weakness. However, despite these challenges, the administration of the cognitive assessment scales proved overall to be feasible in the study sample.

The results indicate that cognitive abilities in developmental age seems to be quantitatively and qualitatively affected in children with NMDs. Consistently with previous reports [13,41], in the study sample DMD patients above 6 years-old and DM1 patients showed lower FSIQ and sub-indices mean scores on WISC-IV scales compared to normative data for age, with a high rate of ID, namely in the DM1 cohort [13,20,42].

On the other hand, in our sample, on the Wechsler IQ scales (i.e., WPSSI-III and WISC-IV), FSIQ and its sub-indices mean scores were not significantly different from the normative mean score in DMD patients below 6 years of age and in BMD, GSD2, HMSN and type 2 or 3 SMA subgroups, which is consistently with the literature [18]. In the type 2 and 3 SMA patients, higher individual scores compared to normative data for age were found, but the small sample size (*n* = 3) and clinical heterogeneity prevented statistical testing. Despite the important limitation, this finding is in general consistent with previous reports that found FSIQ scores in the normal range or above in SMA [32].

It should be highlighted that some tests that contribute to PIQ and PSI of the Wechsler IQ scales, such as symbol search and coding, may be influenced by motor speed and, therefore, muscle function [7]; however, in the study sample these sub-indices mean scores did not appear to be particularly affected in patients with overall lower FSIQ scores (DMD and DM1 patients) or in patients with non-severe weakness (BMD and HSMN patients), even if the heterogeneity and low sample size limit further interpretations.

Of the four children assessed by Bayley-III scales (DM1 *n* = 2, GSD2 *n* = 2), on the Cognitive Scale DM1 patients scored lower than expected for age, while GSD2 patients scored in the normal range, consistent with previous literature report [29]. On the Language Scale, the delay may have been influenced by the fact that there were three bilinguals out of the four examined patients, therefore these results must be confirmed by further investigations with a larger sample size.

Nonetheless, the systematic cognitive assessment design for this study highlighted the presence of early delays, namely in DM1, underlying once again the importance of conducting an early developmental assessment in children with NMDs to identify possible areas of intervention that may improve clinical management and quality of life of patients and their caregivers.

In the DM1 subgroup, a history of delayed language acquisition was associated with the presence of an ID at the time of the assessment. This finding may suggest that an early delay in language, even before the onset of significant muscle weakness, cannot be exclusively attributed to muscle impairment, emotional reaction to illness, or lack of experience opportunities related to motor limitations and, therefore, must prompt cognitive assessments and follow-up in time.

When considering the genotype–phenotype relationship in the sample, no differences in FSIQ scores emerged between proximal and distal dystrophin mutations in the DMD subgroup, but the small sample size provided only limited statistical power.

In the study sample, consistently with previous reports, a greater severity of cognitive impairment was found in the congenital form compared to the infantile one in the DM1 subgroup [23].

Considering the possible associations between cognitive and motor functions, this study found that in the DM1 subgroup, lower WISC-IV FSIQ was associated with lower scores on the motor function assessment scale MFM32, and that, in the DMD cohort aged more than 6 years, lower WISC-FSIQ scores were associated with lower NSAA scores. It is noteworthy, however, that these associations, do not allow cause to be discriminated from effect. It is challenging to establish the direction of the relationship between motor and cognitive function, i.e., whether motor deficits negatively affect the cognitive development of patients or, vice versa, it is the cognitive deficit that limits motor performance. It is likely that in DM1 there is a common pathogenetic mechanism of damage to the neuromuscular system and the CNS.

No significant associations between WISC-IV or WPSSI-III FSIQ and total scores on the motor function scales were found in the DMD cohort aged under 6 years and in the BMD, GSD2, SMA and HMSN subgroups. This result may be again due to the small sample size.

### Limitations

As already acknowledged, the main limitation of this pilot study is the small sample size and a very small number of patients in the NMD subgroups. A second major limitation is the absence of an internal control group. A third limitation is the heterogeneity in disorders age and developmental stage requiring the utilization of different motor and cognitive scales; this heterogeneity constitutes a major limitation that prevents generalizations about the relationship between motor deficit and intellectual functioning in NMDs. A further limitation is that some tests that contribute to PIQ and PSI on the Wechsler scales, such as symbol search and coding, may be influenced by motor speed. Moreover, in various NMD diseases, patients may already show at early developmental age an impairment in vital functions, thus further complicating the heterogeneity of the population and the results. Finally, the lack of neuroradiological data prevents the identification of possible structural brain abnormalities. However, brain MRI examinations are not routinely conducted without specific clinical indications, also because they require pharmacological sedation in young children.

## 5. Conclusions

This study adds to previous findings of several cognitive phenotypes in children and adolescents with NMDs and provides evidence of the feasibility and the importance of a regular and early assessment of cognitive functions in these patients, in order to enable interventions aimed at preserving functioning and quality of life. This seems especially relevant and timely, in the light of the increased survival brought by the recently introduced innovative therapies.

Moreover, a specific focus on cognitive deficits in these disorders can help to reduce the global pathology-related family burden.

Finally, the availability of new therapies, namely in SMA and GSD2, paves the way unexpected developments. This finding should prompt the inclusion of cognitive assessment in the follow-up protocols at visit of specialized tertiary neuromuscular centers.

Further studies with larger samples are needed to characterize trajectories of cognitive, neuropsychological, and adaptive functioning, with a special attention to new phenotypes that are emerging with the availability of innovative therapies.

## Figures and Tables

**Table 1:** Demographics and characteristics of the sample.

Variables	DMD (*n* = 15)	BMD (*n* = 4)	DM1 (*n* = 8)	GSD2 (*n* = 6)	SMA Type 2 or 3 (*n* = 3)	HMSN (*n* = 7)
Males, *n* (%)	15.0 (100)	4.0 (100)	4.0 (50.0)	4.0 (67.0)	2.0 (67.0)	4.0 (57.0)
Age (years), median (IQR or range)						
at symptoms onset	4.0 (2.0)	*	0.0 (8.0)	**	0.5 (0.3–2.0)	7.0 (5.5)
at diagnosis	4.5 (1.5)	7.1 (6.7–7.6)	0.2 (0.3)	(EO) 1.5 month (LO) 4.3 years	***	7.1 (7.5)
at assessment	6.7 (4.3)	12.1 (6.1)	12.9 (6.6)	8.4 (13.0)	10.7 (1.0)	11.5 (5.8)
Family history of NMDs, *n* (%)	9.0 (60.0)	3.0 (100)	-	-	-	6.0 (100)
Resuscitation at birth, *n* (%)	1.0 (7.0)	1 (25.0)	2.0 (25.0)	1.0 (20.0)	0.0 (0.0)	1.0 (14.0)
Hypotonia at birth, *n* (%)	0.0 (0.0)	0.0 (0.0)	5.0 (71.0)	1.0 (25.0)	0.0 (0.0)	0.0 (0.0)
Walking delay, *n* (%)	5.0 (33.0)	1.0 (25.0)	2.0 (40.0)	0.0 (0.0)	****	0.0 (0.0)
Speech delay, *n* (%)	8.0 (53.0)	0.0 (0.0)	5.0 (71.0)	2.0 (33.0)	0.0 (0.0)	1.0 (14.0)
Gestational Age						
Preterm, *n* (%)	2.0 (14.0)	0.0 (0.0)	0.0 (0.0)	2.0 (40.0)	1.0 (33.0)	1.0 (14.0)
Full term, *n* (%)	12.0 (86.0)	4.0 (100)	7.0 (100)	3.0 (60.0)	2.0 (67.0)	6.0 (86.0)

DMD: Duchenne muscular dystrophy; BMD: Becker muscular dystrophy; DM1: myotonic muscular dystrophy type 1; GSD2: glycogen storage disease type 2, SMA: spinal muscular atrophy, type 2 or 3; HMSN: hereditary motor sensory neuropathy, type 1 or 2 diseases; NMDs: Neuromuscular Disorders. Where data were not available for all cases, percentages were calculated on available data. * Two subjects had never presented symptoms referable to muscular dystrophy in their clinical history, while in one case the disease started with asthenia and fatigue at 3 years of age and with episodes of rhabdomyolysis at 6 years, and in another case with fatigue and cramps at 6 years of age. ** Data available for four subjects, two with neonatal onset, one with infantile onset and one with symptoms onset at 8 years of age. *** Age at diagnosis in the two subjects with SMA type 2 was 1 year and 1 month in one and 1 year 7 months in the other, and in the SMA type 3 subject was 2 year and 9 months. **** The SMA type 3 subject acquired autonomous walking at 18 months of age.

**Table 2:** NMDs-related Wechsler Intelligence Scale for Children, Fourth Edition (WISC-IV) sub-indices and full-scale intelligence quotient (FSIQ) scores.

NMD Diagnosis	WISC-IV Scales					
	VCI Mean (SD)	PRI Mean (SD)	WMI Mean (SD)	PSI Mean (SD)	FSIQ Mean (SD) Range	
**DMD (*n* = 8)**	**78.7 (7.7)**	**82.9 (8.4)**	**80.9 (9.7)**	**82.2 (14.2)**	**75.9 (SD 11.1)**	63–94
**BMD (*n* = 4)**	100.5 (8.7)	105.2 (19.0)	109.0 (7.3)	91.0 (2.4)	102.2 (6.9)	95–110
**DM1 (*n* = 6)**	**73.7 (19.2)**	**67. 3 (27.8)**	**70.0 (27.3)**	**66.7 (15.8)**	**61.5 (26.1)**	38–99
**GSD2 (*n* = 4)**	104.7 (8.1)	110.3 (12.0)	95.0 (4.6)	100.0 (5.2)	105 (6.2)	96–110
**SMA (*n* = 3)**	**119.3 (4.1)**	110.0 (9.5)	108.0 (6.9)	98.0 (1.7)	113.3 (4.0)	109–117
**HMSN (*n* = 6)**	99.7 (5.0)	104.7 (7.6)	102.0 (13.9)	98.0 (7.0)	101.7 (6.8)	96–114

VCI: Verbal Comprehension Index; PRI: Perceptual Reasoning Index; WMI: Working Memory Index; PSI: Processing Speed Index; FSIQ: Full Scale IQ; NMD: Neuromuscular Disorders. When appropriate, one sample Z-test was applied for comparison between WISC-IV FSIQ, and sub-indices mean scores between study cohorts and the Italian standardized normative data provided in the WISC-IV test (normative population mean score of 100 with a standard deviation of 15 for FSIQ and sub-indices scores). Statistically significant values (*p* < 0.01, with Bonferroni correction for multiple comparison) are bold.

## Data Availability

Data supporting the findings of this study are available from the corresponding author F.S.R. on request.

## Supplementary Materials

The following are available online at https://www.mdpi.com/article/10.3390/jcm10204777/s1, Table S1. Motor function assessment scales in NMDs, Table S2. Patients’ WPPSI-III scores by NMDs, Table S3. Patients’ WISC-IV scores by NMDs, Table S4. Bayley-III ed. Patients’ Scales Scores.
