# Supplementary material for: Assessing Cognitive Function in Neuromuscular Diseases: A Pilot Study in a Sample of Children and Adolescents
Source: J Clin Med. 2021 Oct 18;10(20):4777. doi: 10.3390/jcm10204777 (PMC8537027; doi:10.3390/jcm10204777)
Supplement: Supplementary file 1 [file jcm-10-04777-s001.zip › jcm-1381316-supplementary.pdf]

Table S1. Motor function assessment scales in NMDs.

Scales	Patients, Age	Function	Description
**MRC (Medical Research Council) Scale for Muscle Strength**	All NMDs, all age	Manual muscle strength testing of six muscle groups (shoulder abduction, elbow flexion, wrist extension, hip flexion, knee extension, and ankle dorsiflexion) on both sides	Scale ranging from 0 (no contraction at all) to 5 (normal) in relation to the maximum expected strength for each examined muscle:<br>- Grade 0: No contraction<br>- Grade 1: Flicker or trace of contraction<br>- Grade 2: Active movement with gravity eliminated<br>- Grade 3: Active movement against gravity<br>- Grade 4: Active movement against gravity and resistance<br>- Grade 5: Normal power<br>Total score ranges from 60 to 0.
**Children's Hospital of Philadelphia Infant Test of Neuromuscular Disorders (CHOP INTEND)**	SMA 1, < 2 years-old	Assessment of motor function by observation of spontaneous movements and elicited reactions	16-items scale, each graded on a scale of 0 to 4 (0 no response, 1 minimal, 2 partial, 3 nearly full, and 4 complete level of response).<br>Total score ranges from 0 to 64.
**Motor Function Measure Test (MFM-32)**	All NMDs, all degrees of disease severity, in both ambulant and non-ambulant, >6 years-old	Measure of severity and progression of motor function	32-item scale, each rated on a 4-point Likert, sub-grouped into 3 domains assessing 3 functional areas:<br>- domain 1 (D1): standing position and transfers (13 items)<br>- domain 2 (D2): axial and proximal motor function (12 items)<br>- domain 3 (D3): distal motor function (7 items).<br>Total score ranges from 0 to 96.
**Motor Function Measure Test (MFM-20)**	All NMDs, all degrees of disease severity, in both ambulant and non-ambulant, 2-6 years-old	Measure severity and progression of motor function	20 of the 32 items of the MFM-32, grouped into sub-scores assessing 3 functional areas close to those obtained for the MFM-32 scale.<br>Total score ranges from 0 to 60.
**North Star Ambulatory Assessment (NSAA)**	Ambulant DMD children > 4 years-old	Measure of functional motor abilities (including standing, getting off the floor, walking, jumping, and running), used to monitor the progression of the disease and treatment effects	17-item rating scale, rated from 0 to 2:<br>- 2 - "Normal" - no obvious modification of activity<br>- 1 - Modified method but achieves goal independent of physical assistance from another<br>- 0 - Unable to achieve independently<br>Total score ranges from 0 (completely non-ambulatory) to 34 (indicating fully-independent function)
**Hammersmith Functional Motor Scale (HFMS)**	Non ambulant/Limited mobility SMA type 2 and 3 children > 30 months-old	Measure of motor function, is ordered in level of item difficulty.	20-item scale, each scored on a 3 point scoring system:<br>- 2 for unaided,<br>- 1 for assistance<br>- 0 for inability.<br>Total score is the sum of all individual items. The total score can range from 0 to 40.
**Hammersmith Functional Motor Scale (HFMS) -Expanded**	Ambulant SMA type 2 and 3, > 30 months-old	Measure of motor function, is ordered in level of item difficulty.	HMFS with 13 additional items (33 items scale) adapted from GMFM (Gross Motor Function Measure). Each item is scored on a 3 point scoring system<br>- 2 for unaided;<br>- 1 for assistance;<br>- 0 for inability.<br>Exceptions are the activities of "lying from sitting" and "lifting the head from prone", in which 1 cannot be scored.

			For each function, increased levels of ability are depicted from left to right.<br>Total score is the sum of all the individual items and can range from 0 to 66.
**Performance of Upper Limb (PUL) Module 1.2**	Weaker ambulatory and non-ambulatory DMD, >5 years-old	Assess upper limb function, with the aim of reflecting the proximal to distal progression of muscle weakness typically observed in DMD	22-item scale, with an entry item to define the starting functional level (which corresponds to the Brooke scale) and 21 items subdivided into shoulder level (4 items), elbow level (9 items), and distal (i.e., wrist and fingers) level (8 items). Each dimension can be scored separately maximum score of:<br>- 16 for the shoulder level,<br>- 34 for the elbow level,<br>- and 24 for the distal level.<br>Total score is the sum of the 3-level scores, with a maximum global score of 74 points.
**Upper Limb Module (ULM)**	Non ambulant SMA type 2 and 3	Assess a number of upper limb activities and axial strength	9-item scale, scored on a 3 point scale using simple criteria:<br>- 2 Normal: achieves goal without any assistance;<br>- 1 Modified method but achieves goal independent of physical assistance from another person;<br>- 0 Unable to achieve independently.<br>Total score is the sum of the individual items and can range from 0 to 18.
**Six Minute Walk Test (6MWT)**	Wide range of ambulant NMDs, preschool children (2-5 years), children (6-12 years) adults (18-64 years), elderly adults (65+)	Sub-maximal exercise test used to assess aerobic capacity and endurance, provides valuable information regarding the natural history of the disorder.	Measured distance covered over a time of 6 minutes on a 25-metres marked course.<br>The result obtained is compared with that expected for age: normal values for a healthy subject under the age of 70 are between 400 and 700 metres.

Table S2. Patients' WPPSI-III scores by NMDs.

ID patient	WPPSI-III Scales			
	**FSIQ scaled score (percentile)**	**VIQ scaled score (percentile)**	**PIQ scaled score (percentile)**	**PSQ scaled score (percentile)**
**DMD-1**	128 (97°)	104 (61°)	122 (93°)	109 (73°)
**DMD-2**	62 (1°)	58 (0,3°)	68 (2°)	61 (0,5°)
**DMD-3**	72 (3°)	64 (1°)	76 (5°)	74 (4°)
**DMD-4**	90 (25°)	88 (21°)	93 (32°)	112 (79°)
**DMD-5**	132 (98°)	120 (91°)	120 (91°)	100 (50°)
**DMD-6**	96 (39°)	104 (61°)	98 (45°)	76 (5°)
**DMD-7**	98 (45°)	104 (61°)	93 (32°)	67 (1°)
**GSD2-3**	107 (68°)	106 (63°)	98 (45°)	119 (90°)
**HMSN-1**	102 (55°)	98 (45°)	104 (61°)	103 (58°)

FSIQ: Full-Scale IQ; VIQ: Verbal IQ; PIQ: Performance IQ; Processing Speed Quotient; DMD: Duchenne Muscular Dystrophy; GSD2: Glycogen Storage Disease Type 2; HMSN: Hereditary Motor Sensory Neuropathy.

Table S3. Patients' WISC-IV scores by NMDs.

ID	WISC-IV Scales				
	**FSIQ scaled score (percentile)**	**VCI scaled score (percentile)**	**PRI scaled score (percentile)**	**WMI scaled score (percentile)**	**PSI scaled score (percentile)**
**DMD-8**	88 (20.5°)	88 (21.4°)	85 (15.5°)	97 (42.8°)	97 (42.4°)
**DMD-9**	94 (34.7°)	84 (15°)	98 (43.8°)	91 (27.9°)	106 (65°)
**DMD-10**	67 (1.3°)	68 (1.5°)	80 (9.6°)	76 (5.5°)	79 (8.9°)
**DMD-11**	72 (3°)	76 (5.8°)	82 (12.3°)	82 (11.5°)	79 (8.9°)
**DMD-12**	82 (11°)	74 (4.3°)	78 (7.7°)	85 (15.9°)	88 (21.5°)
**DMD-13**	66 (1°)	70 (2.2°)	82 (12.3°)	76 (5.5°)	71 (2.3°)
**DMD-14**	75 (4.5°)	86 (18.2°)	89 (23.3°)	70 (1.9°)	76 (6.3°)
**DMD-15**	63 (0.7°)	84 (15°)	69 (2.1°)	70 (1.9°)	62 (0.5°)
**BMD-1**	95 (37.4°)	108 (69.9°)	82 (12.3°)	103 (58.1°)	91 (28°)
**BMD-2**	110 (74°)	104 (59.6°)	117 (86.8°)	118 (88°)	88 (21.5°)
**BMD-3**	106 (66°)	88 (21.4°)	124 (94.3°)	112 (78.6°)	97 (42.4°)
**BMD-4**	98 (45.5°)	102 (54.4°)	98 (43.8°)	103 (58.1°)	91 (28°)
**DM1-3**	85 (15.6°)	100 (49.5°)	93 (32.5°)	82 (11.5°)	74 (4°)
**DM1-4**	99 (47.7°)	92 (29°)	108 (71°)	115 (83.5°)	82 (12.3°)
**DM1-5**	66 (1.1°)	72 (3.1°)	67 (1.4)	79 (8°)	85 (16.6°)
**DM1-6**	42 (<0.1°)	66 (1°)	54 (<0.1°)	46 (<0.1°)	59 (0.3°)
**DM1-7**	38 (<0.1°)	48 (<0.1°)	41 (<0.1°)	46 (<0.1°)	47 (<0.1°)
**DM1-8**	39 (<0.1°)	64 (0.6°)	41 (<0.1)	52 (<0.1°)	53 (<0.1°)
**GSD2-4**	110 (74°)	106 (64.9°)	122 (92.1°)	94 (34.9°)	103 (57.4°)
**GSD2-5**	107 (68.3°)	112 (78.9°)	111 (75.7°)	91 (27.9°)	103 (57.4°)
**GSD2-6**	96 (39.8°)	96 (43.8°)	98 (43.8°)	100 (50.9°)	94 (35.3°)
**SMA-1**	109 (72.4°)	124 (94.4°)	100 (48.8°)	100 (50.9°)	97 (42.4°)
**SMA-2**	117 (87.1°)	116 (86°)	119 (89.6°)	112 (78.6°)	100 (49.8°)
**SMA-3**	114 (81.8°)	118 (88.7°)	111 (80.2°)	112 (78.6°)	97 (42.4°)
**HMSN-2**	96 (39.8°)	100 (49.5°)	100 (48.8°)	76 (5.5°)	109 (71.9°)
**HMSN-3**	97 (43.5°)	94 (33.8°)	98 (43.8°)	103 (58.1°)	97 (42.4°)
**HMSN-4**	98 (45.5°)	98 (44.5°)	102 (54.5°)	106 (65.4°)	88 (21.5°)
**HMSN-5**	114 (81.8°)	108 (69.9°)	119 (89.6°)	118 (88°)	94 (35.3°)
**HMSN-6**	105 (61.9°)	102 (54.4°)	106 (65.9°)	106 (65.4°)	100 (49.8°)
**HMSN-7**	100 (50.2°)	96 (33.1°)	102 (54.5°)	103 (58.1°)	100 (49.8°)

VCI: Verbal Comprehension Index; PRI: Perceptual Reasoning Index; WMI: Working Memory Index; PSI: Processing Speed Index; FSIQ: Full-Scale IQ; DMD: Duchenne Muscular Dystrophy; BMD: Becker Muscular Dystrophy; DM1: Myotonic dystrophy type 1; GSD2: Glycogen Storage Disease Type 2; HMSN: Hereditary Motor Sensory Neuropathy.

Table S4. Bayley-III ed. Patients' Scales Scores.

**DM1-1** *Scale*	*Raw Score*	*Composite Score*	*Percentile*	**DM1-2** *Scale*	*Raw Score*	*Composite Score*	*Percentile*	**GSD2-1** *Scale*	*Raw Score*	*Composite Score*	*Percentile*	**GS2-2** *Scale*	*Raw Score*	*Composite Score*	*Percentile*
**Cognitive**	29	55	0.1°	Cognitive	51	85	16°	Cognitive	50	85	16°	Cognitive	57	95	37°
**Language**	-	65	1°	Language	-	71	3°	Language	-	74	4°	Language	-	79	8°
Receptive	12	-	-	*Receptive*	16	-	-	*Receptive*	15	-	-	*Receptive*	21	-	-
Expressive	13	-		*Expressive*	11	-	-	*Expressive*	18	-	-	*Expressive*	17	-	-
**Motor**	-	49	<0.1°	Motor	-	88	21°	Motor	-	97	42°	Motor	-	70	2°
Fine Motor	22	-	-	*Fine Motor*	36	-	-	*Fine Motor*	37	-	-	*Fine Motor*	36	-	-

Gross Motor	37	-	-	*Gross Motor*	47	-	-	*Gross Motor*	50	-	-	*Gross Motor*	25	-	-
**Socio-Emotional**	65	3	1°	Socio-Emotional	84	80	9°	Socio-Emotional	105	100	50°	Socio-Emotional	122	145	99.9°
**Adaptive**	-	-	-	Adaptive	-	63	1°	Adaptive	-	106	66°	Adaptive	-	63	2°

DM1: Myotonic dystrophy type 1; GSD2: Glycogen Storage Disease Type 2.
